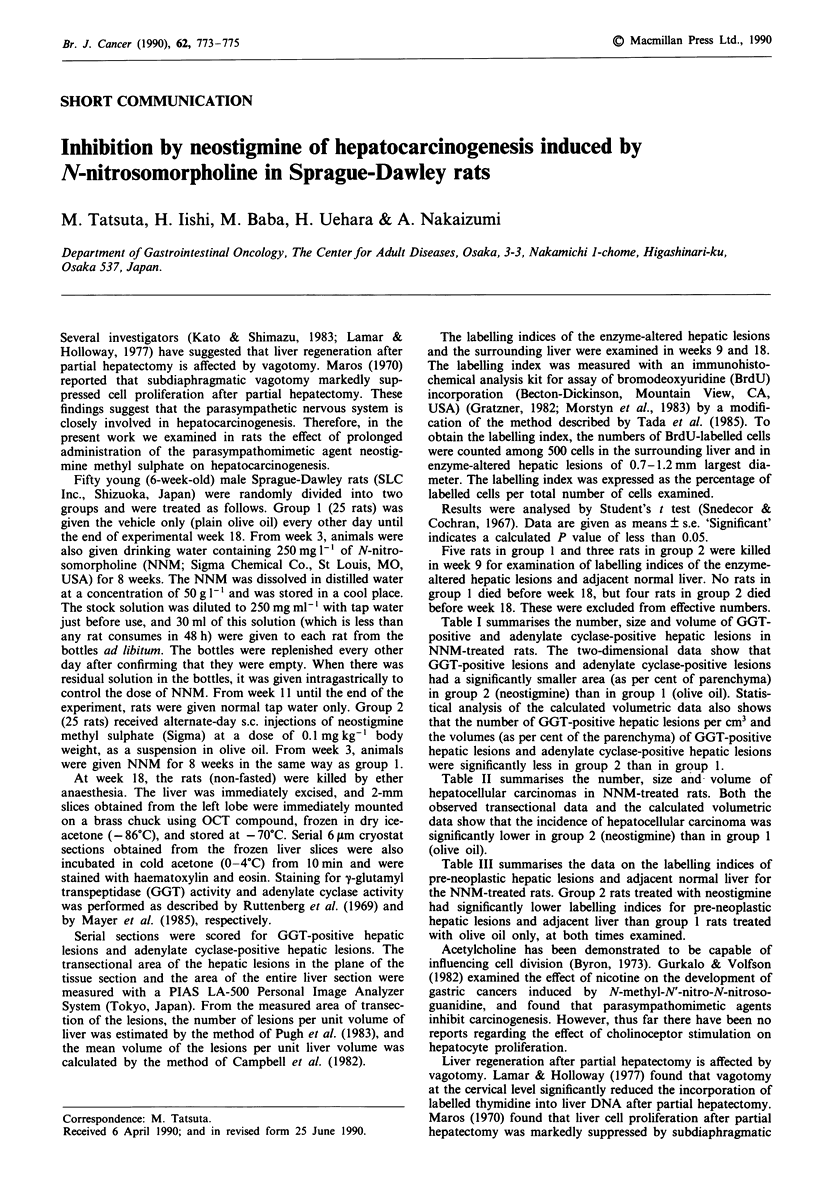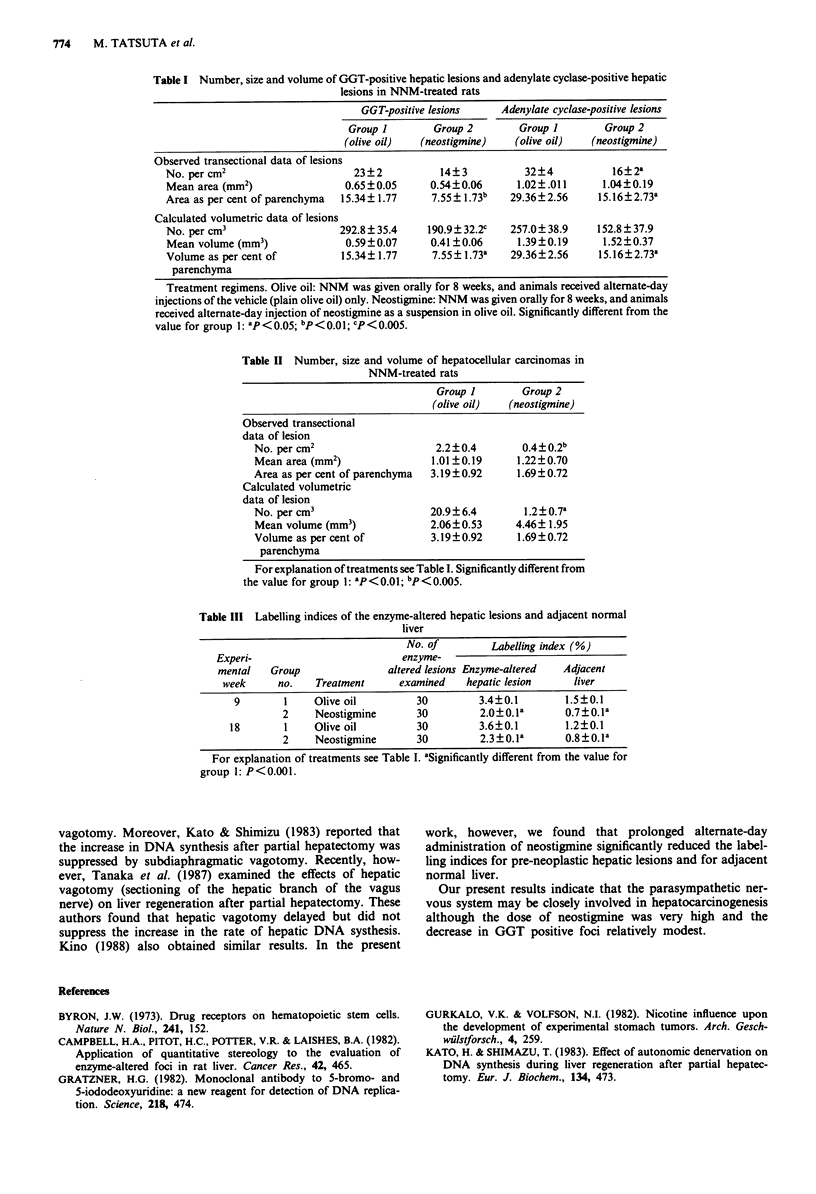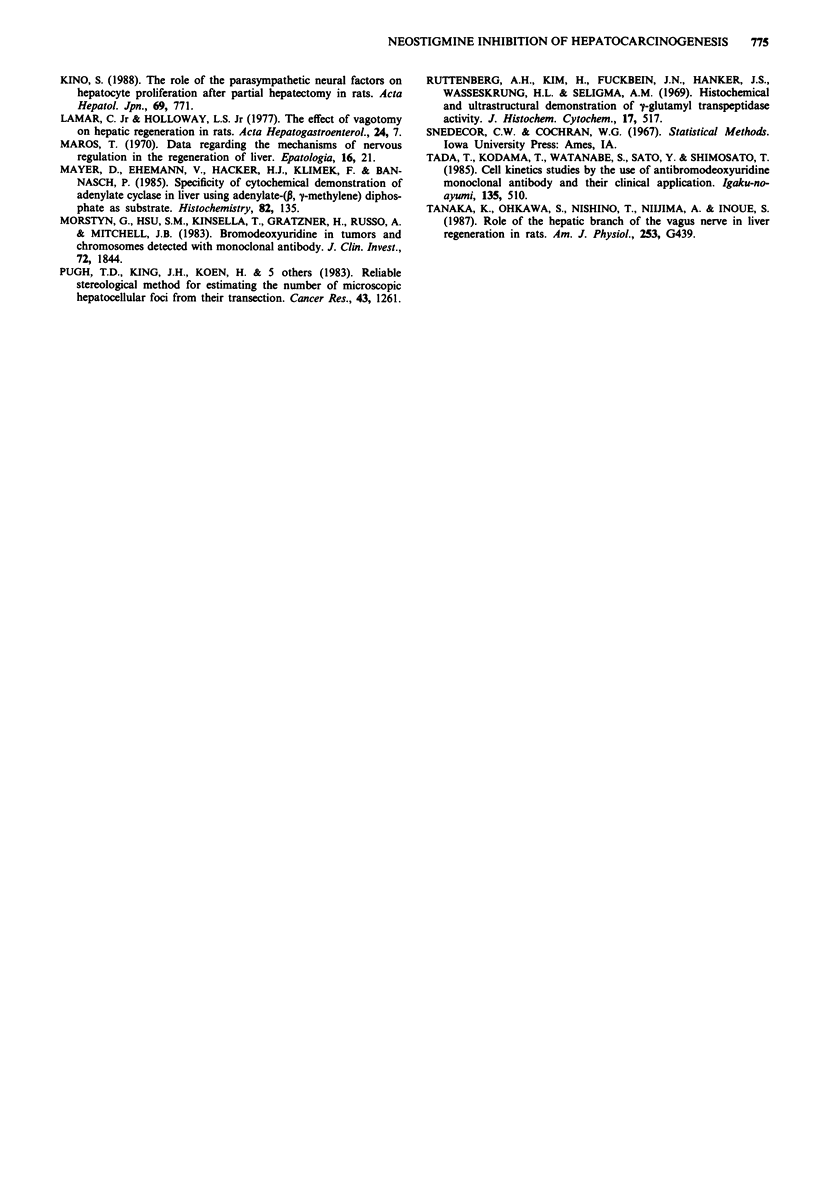# Inhibition by neostigmine of hepatocarcinogenesis induced by N-nitrosomorpholine in Sprague-Dawley rats.

**DOI:** 10.1038/bjc.1990.377

**Published:** 1990-11

**Authors:** M. Tatsuta, H. Iishi, M. Baba, H. Uehara, A. Nakaizumi

**Affiliations:** Department of Gastrointestinal Oncology, Center for Adult Diseases, Osaka, Japan.


Br. J. Cancer (1990), 62, 773-775 © Macmillan Press Ltd., 1990

SHORT COMMUNICATION

### Inhibition by neostigmine of hepatocarcinogenesis induced by N-nitrosomorpholine in Sprague-Dawley rats

M. Tatsuta, H. Iishi, M. Baba, H. Uehara & A. Nakaizumi

*Department of Gastrointestinal Oncology, The Center for Adult Diseases, Osaka, 3-3, Nakamichi 1-chome, Higashinari-ku, Osaka 537, Japan.*

Several investigators (Kato & Shimazu, 1983; Lamar & Holloway, 1977) have suggested that liver regeneration after partial hepatectomy is affected by vagotomy. Maros (1970) reported that subdiaphragmatic vagotomy markedly suppressed cell proliferation after partial hepatectomy. These findings suggest that the parasympathetic nervous system is closely involved in hepatocarcinogenesis. Therefore, in the present work we examined in rats the effect of prolonged administration of the parasympathomimetic agent neostigmine methyl sulphate on hepatocarcinogenesis.

Fifty young (6-week-old) male Sprague-Dawley rats (SLC Inc., Shizuoka, Japan) were randomly divided into two groups and were treated as follows. Group 1 (25 rats) was given the vehicle only (plain olive oil) every other day until the end of experimental week 18. From week 3, animals were also given drinking water containing 250 mg $l^{-1}$ of N-nitrosomorpholine (NNM; Sigma Chemical Co., St Louis, MO, USA) for 8 weeks. The NNM was dissolved in distilled water at a concentration of 50 g $l^{-1}$ and was stored in a cool place. The stock solution was diluted to 250 mg $ml^{-1}$ with tap water just before use, and 30 ml of this solution (which is less than any rat consumes in 48 h) were given to each rat from the bottles *ad libitum*. The bottles were replenished every other day after confirming that they were empty. When there was residual solution in the bottles, it was given intragastrically to control the dose of NNM. From week 11 until the end of the experiment, rats were given normal tap water only. Group 2 (25 rats) received alternate-day s.c. injections of neostigmine methyl sulphate (Sigma) at a dose of 0.1 mg $kg^{-1}$ body weight, as a suspension in olive oil. From week 3, animals were given NNM for 8 weeks in the same way as group 1.

At week 18, the rats (non-fasted) were killed by ether anaesthesia. The liver was immediately excised, and 2-mm slices obtained from the left lobe were immediately mounted on a brass chuck using OCT compound, frozen in dry ice-acetone (−86°C), and stored at −70°C. Serial 6 µm cryostat sections obtained from the frozen liver slices were also incubated in cold acetone (0–4°C) from 10 min and were stained with haematoxylin and eosin. Staining for γ-glutamyl transpeptidase (GGT) activity and adenylate cyclase activity was performed as described by Ruttenberg *et al.* (1969) and by Mayer *et al.* (1985), respectively.

Serial sections were scored for GGT-positive hepatic lesions and adenylate cyclase-positive hepatic lesions. The transectional area of the hepatic lesions in the plane of the tissue section and the area of the entire liver section were measured with a PIAS LA-500 Personal Image Analyzer System (Tokyo, Japan). From the measured area of transection of the lesions, the number of lesions per unit volume of liver was estimated by the method of Pugh *et al.* (1983), and the mean volume of the lesions per unit liver volume was calculated by the method of Campbell *et al.* (1982).

The labelling indices of the enzyme-altered hepatic lesions and the surrounding liver were examined in weeks 9 and 18. The labelling index was measured with an immunohistochemical analysis kit for assay of bromodeoxyuridine (BrdU) incorporation (Becton-Dickinson, Mountain View, CA, USA) (Gratzner, 1982; Morstyn *et al.*, 1983) by a modification of the method described by Tada *et al.* (1985). To obtain the labelling index, the numbers of BrdU-labelled cells were counted among 500 cells in the surrounding liver and in enzyme-altered hepatic lesions of 0.7–1.2 mm largest diameter. The labelling index was expressed as the percentage of labelled cells per total number of cells examined.

Results were analysed by Student's *t* test (Snedecor & Cochran, 1967). Data are given as means ± s.e. 'Significant' indicates a calculated *P* value of less than 0.05.

Five rats in group 1 and three rats in group 2 were killed in week 9 for examination of labelling indices of the enzyme-altered hepatic lesions and adjacent normal liver. No rats in group 1 died before week 18, but four rats in group 2 died before week 18. These were excluded from effective numbers.

Table I summarises the number, size and volume of GGT-positive and adenylate cyclase-positive hepatic lesions in NNM-treated rats. The two-dimensional data show that GGT-positive lesions and adenylate cyclase-positive lesions had a significantly smaller area (as per cent of parenchyma) in group 2 (neostigmine) than in group 1 (olive oil). Statistical analysis of the calculated volumetric data also shows that the number of GGT-positive hepatic lesions per $cm^3$ and the volumes (as per cent of the parenchyma) of GGT-positive hepatic lesions and adenylate cyclase-positive hepatic lesions were significantly less in group 2 than in group 1.

Table II summarises the number, size and volume of hepatocellular carcinomas in NNM-treated rats. Both the observed transectional data and the calculated volumetric data show that the incidence of hepatocellular carcinoma was significantly lower in group 2 (neostigmine) than in group 1 (olive oil).

Table III summarises the data on the labelling indices of pre-neoplastic hepatic lesions and adjacent normal liver for the NNM-treated rats. Group 2 rats treated with neostigmine had significantly lower labelling indices for pre-neoplastic hepatic lesions and adjacent liver than group 1 rats treated with olive oil only, at both times examined.

Acetylcholine has been demonstrated to be capable of influencing cell division (Byron, 1973). Gurkalo & Volfson (1982) examined the effect of nicotine on the development of gastric cancers induced by N-methyl-N′-nitro-N-nitrosoguanidine, and found that parasympathomimetic agents inhibit carcinogenesis. However, thus far there have been no reports regarding the effect of cholinoceptor stimulation on hepatocyte proliferation.

Liver regeneration after partial hepatectomy is affected by vagotomy. Lamar & Holloway (1977) found that vagotomy at the cervical level significantly reduced the incorporation of labelled thymidine into liver DNA after partial hepatectomy. Maros (1970) found that liver cell proliferation after partial hepatectomy was markedly suppressed by subdiaphragmatic

Correspondence: M. Tatsuta.
Received 6 April 1990; and in revised form 25 June 1990.

**Table I** Number, size and volume of GGT-positive hepatic lesions and adenylate cyclase-positive hepatic lesions in NNM-treated rats

| | *GGT-positive lesions* | | *Adenylate cyclase-positive lesions* | |
|---|---|---|---|---|
| | *Group 1 (olive oil)* | *Group 2 (neostigmine)* | *Group 1 (olive oil)* | *Group 2 (neostigmine)* |
| Observed transectional data of lesions | | | | |
| No. per $cm^2$ | 23±2 | 14±3 | 32±4 | 16±2[a] |
| Mean area ($mm^2$) | 0.65±0.05 | 0.54±0.06 | 1.02±.011 | 1.04±0.19 |
| Area as per cent of parenchyma | 15.34±1.77 | 7.55±1.73[b] | 29.36±2.56 | 15.16±2.73[a] |
| Calculated volumetric data of lesions | | | | |
| No. per $cm^3$ | 292.8±35.4 | 190.9±32.2[c] | 257.0±38.9 | 152.8±37.9 |
| Mean volume ($mm^3$) | 0.59±0.07 | 0.41±0.06 | 1.39±0.19 | 1.52±0.37 |
| Volume as per cent of parenchyma | 15.34±1.77 | 7.55±1.73[a] | 29.36±2.56 | 15.16±2.73[a] |

Treatment regimens. Olive oil: NNM was given orally for 8 weeks, and animals received alternate-day injections of the vehicle (plain olive oil) only. Neostigmine: NNM was given orally for 8 weeks, and animals received alternate-day injection of neostigmine as a suspension in olive oil. Significantly different from the value for group 1: [a]$P<0.05$; [b]$P<0.01$; [c]$P<0.005$.

**Table II** Number, size and volume of hepatocellular carcinomas in NNM-treated rats

| | *Group 1 (olive oil)* | *Group 2 (neostigmine)* |
|---|---|---|
| Observed transectional data of lesion | | |
| No. per $cm^2$ | 2.2±0.4 | 0.4±0.2[b] |
| Mean area ($mm^2$) | 1.01±0.19 | 1.22±0.70 |
| Area as per cent of parenchyma | 3.19±0.92 | 1.69±0.72 |
| Calculated volumetric data of lesion | | |
| No. per $cm^3$ | 20.9±6.4 | 1.2±0.7[a] |
| Mean volume ($mm^3$) | 2.06±0.53 | 4.46±1.95 |
| Volume as per cent of parenchyma | 3.19±0.92 | 1.69±0.72 |

For explanation of treatments see Table I. Significantly different from the value for group 1: [a]$P<0.01$; [b]$P<0.005$.

**Table III** Labelling indices of the enzyme-altered hepatic lesions and adjacent normal liver

| *Experimental week* | *Group no.* | *Treatment* | *No. of enzyme-altered lesions examined* | *Labelling index (%)* Enzyme-altered hepatic lesion | *Labelling index (%)* Adjacent liver |
|---|---|---|---|---|---|
| 9 | 1 | Olive oil | 30 | 3.4±0.1 | 1.5±0.1 |
| | 2 | Neostigmine | 30 | 2.0±0.1[a] | 0.7±0.1[a] |
| 18 | 1 | Olive oil | 30 | 3.6±0.1 | 1.2±0.1 |
| | 2 | Neostigmine | 30 | 2.3±0.1[a] | 0.8±0.1[a] |

For explanation of treatments see Table I. [a]Significantly different from the value for group 1: $P<0.001$.

vagotomy. Moreover, Kato & Shimizu (1983) reported that the increase in DNA synthesis after partial hepatectomy was suppressed by subdiaphragmatic vagotomy. Recently, however, Tanaka et al. (1987) examined the effects of hepatic vagotomy (sectioning of the hepatic branch of the vagus nerve) on liver regeneration after partial hepatectomy. These authors found that hepatic vagotomy delayed but did not suppress the increase in the rate of hepatic DNA systhesis. Kino (1988) also obtained similar results. In the present work, however, we found that prolonged alternate-day administration of neostigmine significantly reduced the labelling indices for pre-neoplastic hepatic lesions and for adjacent normal liver.

Our present results indicate that the parasympathetic nervous system may be closely involved in hepatocarcinogenesis although the dose of neostigmine was very high and the decrease in GGT positive foci relatively modest.

### References

BYRON, J.W. (1973). Drug receptors on hematopoietic stem cells. *Nature N. Biol.*, **241,** 152.

CAMPBELL, H.A., PITOT, H.C., POTTER, V.R. & LAISHES, B.A. (1982). Application of quantitative stereology to the evaluation of enzyme-altered foci in rat liver. *Cancer Res.*, **42,** 465.

GRATZNER, H.G. (1982). Monoclonal antibody to 5-bromo- and 5-iododeoxyuridine: a new reagent for detection of DNA replication. *Science*, **218,** 474.

GURKALO, V.K. & VOLFSON, N.I. (1982). Nicotine influence upon the development of experimental stomach tumors. *Arch. Geschwülstforsch.*, **4,** 259.

KATO, H. & SHIMAZU, T. (1983). Effect of autonomic denervation on DNA synthesis during liver regeneration after partial hepatectomy. *Eur. J. Biochem.*, **134,** 473.

KINO, S. (1988). The role of the parasympathetic neural factors on hepatocyte proliferation after partial hepatectomy in rats. *Acta Hepatol. Jpn.*, **69**, 771.

LAMAR, C. Jr & HOLLOWAY, L.S. Jr (1977). The effect of vagotomy on hepatic regeneration in rats. *Acta Hepatogastroenterol.*, **24**, 7.

MAROS, T. (1970). Data regarding the mechanisms of nervous regulation in the regeneration of liver. *Epatologia*, **16**, 21.

MAYER, D., EHEMANN, V., HACKER, H.J., KLIMEK, F. & BANNASCH, P. (1985). Specificity of cytochemical demonstration of adenylate cyclase in liver using adenylate-(β, γ-methylene) diphosphate as substrate. *Histochemistry*, **82**, 135.

MORSTYN, G., HSU, S.M., KINSELLA, T., GRATZNER, H., RUSSO, A. & MITCHELL, J.B. (1983). Bromodeoxyuridine in tumors and chromosomes detected with monoclonal antibody. *J. Clin. Invest.*, **72**, 1844.

PUGH, T.D., KING, J.H., KOEN, H. & 5 others (1983). Reliable stereological method for estimating the number of microscopic hepatocellular foci from their transection. *Cancer Res.*, **43**, 1261.

RUTTENBERG, A.H., KIM, H., FUCKBEIN, J.N., HANKER, J.S., WASSESKRUNG, H.L. & SELIGMA, A.M. (1969). Histochemical and ultrastructural demonstration of γ-glutamyl transpeptidase activity. *J. Histochem. Cytochem.*, **17**, 517.

SNEDECOR, C.W. & COCHRAN, W.G. (1967). *Statistical Methods.* Iowa University Press: Ames, IA.

TADA, T., KODAMA, T., WATANABE, S., SATO, Y. & SHIMOSATO, T. (1985). Cell kinetics studies by the use of antibromodeoxyuridine monoclonal antibody and their clinical application. *Igaku-no-ayumi*, **135**, 510.

TANAKA, K., OHKAWA, S., NISHINO, T., NIIJIMA, A. & INOUE, S. (1987). Role of the hepatic branch of the vagus nerve in liver regeneration in rats. *Am. J. Physiol.*, **253**, G439.